# Nurse-led navigation to provide early palliative care in rural areas: a pilot study

**DOI:** 10.1186/s12904-017-0211-2

**Published:** 2017-06-05

**Authors:** Barbara Pesut, Brenda Hooper, Marnie Jacobsen, Barbara Nielsen, Miranda Falk, Brian P. O ‘Connor

**Affiliations:** 1 School of Nursing, University of British Columbia, Okanagan, 1147 Research Road, Kelowna, BC V1V 1V7 Canada; 2 Greater Trail Hospice Society, 1500 Columbia Ave, Suite 7, Rossland, BC V1R 1J9 Canada; 3 Quesnel Primary Care Clinic, Quesnel, BC Canada; 4 School of Nursing, University of British Columbia, Okanagan, 1147 Research Road, Kelowna, BC V1V 1V7 Canada; 5 Department of Psychology, University of British Columbia, Okanagan, 1147 Research Road, Kelowna, BC V1V 1V7 Canada

**Keywords:** Rural health services, Chronic disease, Palliative care, Patient navigation, Nursing, Palliative approach

## Abstract

**Background:**

Few services are available to support rural older adults living at home with advancing chronic illness. The objective of this project was to pilot a nurse-led navigation service to provide early palliative support for rural older adults and their families living at home with advancing chronic illness.

**Methods:**

Twenty-five older adults and 11 family members living with advancing chronic illness received bi-weekly home visits by a nurse navigator over a 2-year period. Navigation services included symptom management, education, advance care planning, advocacy, mobilization of resources, and psychosocial support. The nurse navigator collected longitudinal data on older adult and family needs, and older adult quality of life and healthcare utilization.

**Results:**

Satisfaction with the service was high. There was no attrition over the 2-year period except through death, and few cancelled visits, indicating a high degree of acceptability of the intervention. The navigator addressed complex, multi-faceted needs through connecting health, social, and informal community resources. Participants who indicated a preferred place of death were able to die in that preferred place (*n* = 7). Emergency room use by participants was minimal and largely unpreventable by the nurse navigator. Longitudinal health-related quality of life scores for many participants were poor, lending further support to the need for more focused attention to this upstream palliative population.

**Conclusions:**

Using a nurse navigator to facilitate early palliative care for rural older adults living with advanced chronic illness is a promising innovation for meeting the needs of this population. Further research is required to evaluate outcomes on a larger scale.

## Background

Finding innovative ways to care for a population aging with complex, chronic illness is high on the healthcare policy agenda [1, 2]. Of particular concern is the need to close gaps in support for those transitioning from chronic illness management to palliative care [3, 4], a time of advancing chronic illness. This is an early palliative population; they are not imminently dying, but death within a year would not come as a surprise [5]. A palliative approach has been used to describe ideal care for this population [6–8]. However, little evidence exists on how to realize this ideal [9]. A palliative approach is defined as palliative care that is adapted to non-specialist contexts, chronic disease conditions, and provided early in the palliative trajectory with the intent to ease the transition between chronic disease management and end-of-life care [10].

Evidence describing the challenges facing older adults living at home with advanced chronic illness is compelling [11, 12], illustrating the urgent need for enhanced support. This population lives with heavy symptom burden [13–15] and are at risk for social isolation [16]. They have high needs for support, anticipatory guidance, advocacy, and assistance with decision-making [17]; however, they may not yet be eligible for home-based nursing services, which in Canada are often provided later in the palliative trajectory. Further, older adults and family are often unaware of the health and social services available to them in their community [18]. Indeed, because of a lack of suitable supports, this time on the palliative trajectory may be more problematic than the actively dying phase [19].

Rurality adds a layer of complexity to this already challenging picture. Rural healthcare services are limited [20] and inaccessible, in part, because of personnel shortages [21]. As a result, rural persons living with a palliative diagnosis undergo more transitions in care than their urban counterparts [22]. However, rural communities also have unique capacity to provide high quality care [23]. The inherent accountability that arises from caring for those for whom personal and professional relationships co-exist, and the commitment of local palliative champions, support good care [24]. As such, the solutions to rural healthcare service limitations often lie in building this inherent rural community capacity. This in turn supports the ideal of aging and dying in place for rural individuals.

In keeping with this community-capacity building approach, the aim of this study was to pilot a nurse-led, rural early palliative service. Individuals living with advanced chronic illness received in-home visits by a nurse who performed a supportive navigation role. A previous publication provided data on community-capacity building strategies in preparation for the service and early feasibility findings [25]. This publication provides summative evaluation findings.

## Methods

This was a single-centre, 2-year (2013–2014) observational study. Specifically, we sought to answer three questions: Is the service feasible, and if so, what is the optimal visit schedule, navigator to client ratio, and specific nature of the services provided? What is the acceptability of the service for all stakeholders? What are preliminary outcomes of the intervention on older adult quality of life, family needs, and healthcare utilization? Evaluation data was collected using mixed method approaches. After ethical approval by the university and health authority, older adults living with advanced chronic illness and a family member, if applicable, were recruited to participate. All participants provided written, informed consent. Older adults were visited in the home by the nurse navigator who provided symptom management, education, advance care planning, advocacy, mobilization of resources, and psychosocial support. The role resembled that of a nurse navigator described in the literature [26–28]. However, the navigation intervention entailed more intensive contact than is typically provided by either navigators or case managers and focused beyond formal healthcare services to include community social care and informal networks. The nurse navigator was supported by a nurse practitioner and general practitioner with palliative expertise; monthly meetings were held to discuss participants’ progress. The nurse practitioner provided coverage when the nurse navigator was not available (e.g., holidays, illness). Participants were provided with an on-call line, available 24/7, for assistance between visits. To ensure good communication with the primary care physician and other healthcare providers, participants signed an information sharing consent. Primary care physicians received regular faxes about relevant clinical issues. Telephone consultation was used for more pressing clinical issues. Older adults were provided with an in-home journal that detailed relevant issues to share with their other care providers.

### Setting and participants

The study was conducted across two rural communities with populations of under 10,000, located 30 min apart by car. Healthcare services included a shared hospital and limited home care services, but no specialized palliative services or hospice beds. In the Canadian context, specialized services mean the availability of a multi-disciplinary palliative team and dedicated palliative beds. Further, in rural areas limited home care services are typically reserved for those patients who require specific nursing tasks (e.g., wound management). The nurse navigator had a graduate degree in counseling, extensive clinical experience in home-based palliative care, and had lived and worked in the rural community for most of her career. Participants were 25 older adults living with advanced chronic illness (see Table 1) who were recruited through public advertising (*n* = 10) and healthcare providers (*n* = 15). Recruitment was ongoing to keep enrollment at capacity after attrition due to death. Eligibility criteria were adults, aged 55 or older, living with one or more chronic illnesses that could reasonably lead to death within the next year [5]. Exclusion criteria included dementia because this population within this rural community was likely to be institutionalized in their final year of life. In addition to the 25 recruited, 25 were screened but did not participate. Fifteen declined participation after receiving further information. Ten were deemed ineligible; reasons included too close to death and already had services in the home (*n* = 5); dementia (*n* = 2); and condition not life-limiting (*n* = 3). Eleven family members were recruited, 8 partners and 3 adult children.

**Table 1:** Demographic information for study participants

Sex	Female: *n* = 11 Male: *n* = 14
Age on enrollment	Range: 57–93 Mean (SD): 74.12 (10.27)
Living arrangements	At home: *n* = 6 At home with family: *n* = 17 In supportive living: *n* = 2
Time on study (in days)	*Alive at study conclusion: n = 14* Range: 54–664 Mean (SD): 421.07 (193.68) *Deceased: n = 11* Range: 30–628 Mean (SD): 246.45 (213.53)
Participant primary diagnosis	Cancer: *n* = 13 Heart Failure: *n* = 4 COPD: *n* = 1 Neurodegenerative: *n* = 1 Other: *n* = 6

### Data collection and analysis

At each scheduled visit the nurse navigator recorded data on visit characteristics (e.g., length, type, persons present), needs and services provided, participant quality of life (QOL) using the McGill QOL Questionnaire (MQOL) [29, 30], family needs using the Caregiver Support Needs Survey (CSNS) [31, 32], and healthcare utilization since the previous visit. The MQOL is a 17-item instrument measuring both overall and health-related quality of life using physical, psychosocial and existential subscales. The CSNS asks participants to rate 25 common caregiver tasks on two dimensions, importance of the need, and the degree to which it is being met. Administration schedules varied as participants completed questionnaires only when they felt well enough to do so. Visit characteristics were recorded using a structured reporting form that required the navigator to fill in needs identified and care provided under six domains. Healthcare utilization was recorded on the research report using a tick-box of available services. Quantitative was managed using SPSS Software ^IBM^.

Data regarding acceptability and satisfaction were collected from older adults who remained alive at study conclusion, family members, and other stakeholders (e.g., advisory group, physicians, health region decision makers) using semi-structured interviews. Interviews were conducted by the principal investigator or trained research assistant either in person or by telephone. An interview guide was used to gather data on experiences with the service. Interviews were audio-taped, transcribed verbatim, and analyzed using NVIVO^QSR^ software. A thematic analysis [33] was constructed based upon the research questions of experiences of the service, services provided, satisfaction with services, and advice for improvements.

## Results

Feasibility, acceptability and outcomes are reported through the services provided, longitudinal MQOL and CSNS scores, healthcare utilization, and qualitative evaluation interviews.

### Services provided

Participants were visited weekly during the first 9 months of the study and then biweekly for the remainder of the study. This change was implemented to increase service capacity. Visit schedules were flexible to accommodate participant needs. Table 2 provides an overview of the visits conducted over the 2-year period. This flexible approach resulted in only 28 (0.05%) cancelled visits over the study period. Half of these were cancelled by 2 participants who were experiencing heavy symptom burden. There was no study attrition, except through death; one participant declined visits for 6 months and then resumed participation. Mean visit duration of scheduled, in-person visits, even for long-term participants, remained stable at approximately 1 h. This time was necessary to address the complex challenges participants were experiencing, while respecting the hospitality central to rural relationships (e.g., the requisite cup of tea). Unscheduled visits occurred when either the nurse navigator, participant, or family member identified a need that required attention beyond the regularly scheduled visit. These unscheduled visits represented 20% of the total visits, of which 38% were initiated by study participants. The on-call line was used 47 times over the 2-year period by a participant or family member; participants indicated that their needs were well met through the regular visits.

**Table 2:** Visit characteristics

Total contacts	*N* = 631^a^ In person: *n* = 553, By telephone: *n* = 78	
Length of visit by nature and person present.	Visit method and persons present	Length, in minutes Mean (SD)
	In person with participant only: *n* = 326	57 (23)
	In person with participant dyads only: *n* = 77	85 (37)
	Telephone with participant only: *n* = 37	10 (3)
	In person with family caregiver only: *n* = 7	49 (27)
	Telephone with family caregiver only: *n* = 26	9 (3)
	In person with both participant and family caregiver: *n* = 137	67 (21)
	Telephone with both participant and family caregiver: *n* = 6	10 (0)
	In person with other family (non-registrant): *n* = 6	18 (9)
	Telephone with other family (non-registrant): *n* = 9	13 (4)
Scheduled versus Unscheduled	Scheduled: *n* = 508 Unscheduled: *n* = 123 of which 76 (62%) initiated by navigator	
Use of 24/7 call line	By participant: *n* = 19, 4 resulted in home visit By family/friend: *n* = 28, 8 resulted in home visit Phone call from other family/friend: *n* = 8, 2 resulted in home visit	

^a^This number excludes bereavement visits with family

Data revealed the multi-faceted problems (e.g., family conflict, financial challenges, troubling symptoms, mobility issues) with which these participants were coping. The nurse navigator addressed these challenges incrementally over subsequent visits. Part of her role included bridging the gaps between health and social care. For example, one older adult had a persistent environmental problem, which was impacting his health, that required several months of negotiation by the navigator with agencies and contractors. Table 3 provides examples of typical interventions performed by the nurse navigator. Primary interventions were teaching about symptom management and psychosocial support for the emotional challenges characteristic of living with advanced illness. Assisting older adults to comprehend healthcare information and make decisions about care was also central to the role.

**Table 3:** Examples of nurse navigator interventions

Domain of support^a^	Examples of interventions
Disease management	Teaching about disease treatment, trajectories, medication and side effect management. Coaching regarding communicating with healthcare providers and healthcare utilization. Accessing disease management resources in the community, at tertiary treatment centres, and online. Discussing decisions regarding treatment choices.
Spiritual	Conversations about fear of dying, spiritual guidance, negative religious coping, afterlife, suffering, involvement in church. Referrals to community chaplain. Life reminiscing and dignity therapy.
Physical	Teaching and assistance with managing common symptoms such as fatigue, pain, mobility limitations, skin irritation, shortness of breath, and bowel and bladder problems. Referrals to healthcare services. Falls prevention strategies.
Practical	Obtaining equipment from Red Cross Loan Cupboard. Mobilizing assistance for transportation, meals, housekeeping, and assistance with ADLs.
Psychological	Support for concerns such as anxiety, depression, stress, and grief. Practical interventions to attenuate psychological concerns (e.g., stress management strategies and art therapy). Referrals to family physician or mental health services.
EOL	Advance care planning including funeral arrangements, planning regarding place of death, representation agreements, palliative benefits, wills. Dignity therapy. Teaching on what to expect at end of life. Support during last days at home.
Social	Negotiating family challenges. Facilitating connections to supportive networks. Strategies to cope with social isolation. Providing resources when commuting outside of rural area for care.
Loss and Grief	Supporting through anticipatory grief and into the bereavement period. Practical strategies to cope with multiple losses. Attended funerals.

^a^Domains were developed based upon A Model to Guide Hospice Care by the Canadian Hospice Palliative Care Association

### Participant QOL and family needs scores

**Fig. 1:**
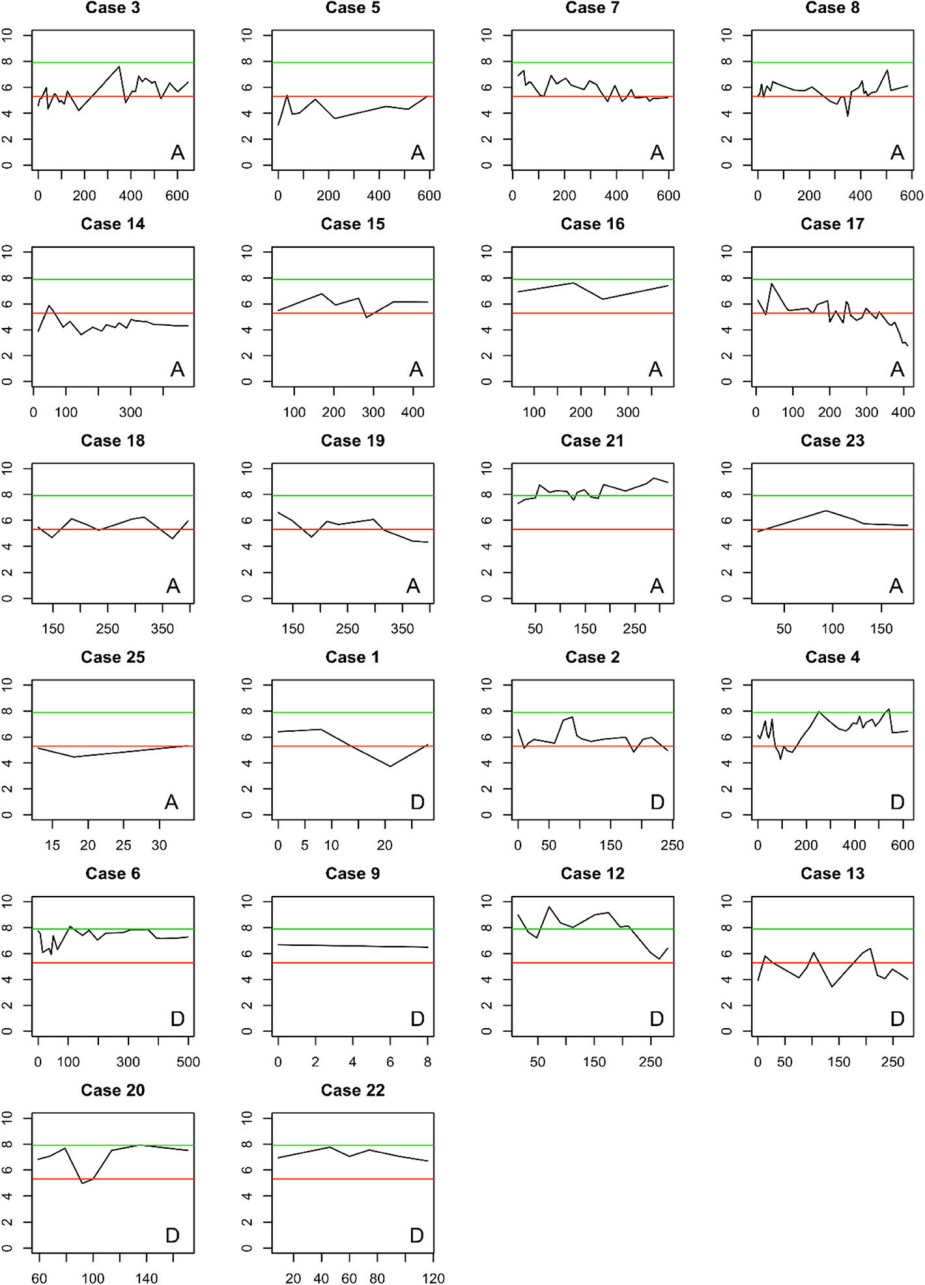
Quality of Life Scores (x) for Older Adult Participants over Days on Service (y). Three participants did not complete MQOL scores. *A* = Alive at study conclusion *D* = Deceased at study conclusion. *Green line* indicates a score that would be consider a “good day” and *red line* indicates a score that would be considered a “bad day” [34]

Longitudinal collection of older adult QOL and family needs served two purposes: to describe outcomes and their trajectories over time and to act as a patient-reported outcome measure to guide clinical care. Figure 1 illustrates longitudinal scores of health-related QOL (scores calculated from five sub-domains) for participants. Overlaying lines provide norm-referenced scores derived from another study [34] that describe a “good day” (means of 7.9) and a “bad day” (means of 5.3). Trajectories reflected a stable pattern for most participants. Four participant graphs illustrate declining scores over the study period; two illustrate mildly improved scores.

Obtaining high quality data on the Caregiver Support Needs Survey was challenging. Over subsequent administrations, 14.7% of total data was missing, rendering many of the questionnaires unusable for analysis. Nine of 11 family members provided valid data on admission to the study. Top four unmet needs were related to information support: *receiving information on ways of comforting the patient* (*n* = 5); *receiving information on patient’s psychological needs* (*n* = 4); *receiving information on symptoms* (*n* = 3); and *receiving information on activities and exercises for the patient* (*n* = 3). Seven family members produced usable questionnaires whereby data could be compared over two time points, the first administration and the last administration (Table 4). Families of patients who died prior to study conclusion reported increasing unmet needs in information, tangible support and emotional support. Families of patients who were alive at study conclusion, reported better information and tangible support scores at the second measurement interval.

**Table 4:** Family need scores over two time points

	Information support needs (max 172) Mean (SD)	Tangible support needs (max 112) Mean (SD)	Emotional support needs (max 112) Mean (SD)
T1, All	59.29 (22.52)	23.57 (13.34)	26.75 (6.02)
T2, All	58.14 (23.49)	22.29 (16.30)	27.86 (10.84)
T1, Deceased	54.00 (35.37)	10.67 (1.15)	25.67 (9.50)
T2, Deceased	77.67 (15.95)	19.67 (13.50)	26.33 (12.01)
T1, Alive at study conclusion	63.25 (11.48)	33.25 (7.97)	27.56 (3.20)
T2, Alive at study conclusion	43.50 (16.34)	24.25 (19.94)	29.00 (11.60)
Comparative rural sample from another study [46]	55.8 (25.4)	28.3 (18.3)	26.9 (12.9)

Higher scores indicate more unmet needs. The maximum values represent worst possible scores

Time 1 (T1) and Time 2 (T2) are first and last usable scores. The length of time between T1 and T2 varied between participants

Experiences of using the MQOL and CSNS were collected in the evaluation interviews. Use of the MQOL was well received once participants became comfortable with the personal nature of the questions. Participants suggested that the questionnaire facilitated open conversations and enabled them to reflect on their lives in a way they might not otherwise have done. Others suggested that some of the questions were not relevant and indicated that it should be done less frequently. Challenges getting quality data on the CSNS were explained in the evaluation interviews. Family members indicated confusion about whether they were filling it out correctly. For example, if they felt a need was better addressed by someone other than the nurse navigator (e.g., spiritual needs), they were not certain how to answer. One participant was reluctant to answer the questions because they “forced her into things she did not want to say.”

### Place of death and healthcare utilization

All participants who indicated a preferred place of death (7/11) were able to die in that preferred place, 5 at home and 2 in institutional settings. Emergency room usage for participants was minimal (see Table 5). Of the 11 decedents, 4 recorded no use of the emergency room, and 3 recorded one use. Importantly, only 6 of the 64 total emergency room admissions could have been prevented by the nurse navigator if she had been contacted (e.g., skin tears and constipation). Those participants who died while on the service reported more physician visits and hospitalizations than those who remained alive at study conclusion. Over the study period, these participants were involved with 23 different physician specialities and a total of 33 different healthcare related groups (e.g., physicians, physician specialties, allied health personnel, and volunteers). An important role of the nurse navigator was to promote continuity for these participants and to assist them to make sense of the information that came from these many providers.

**Table 5:** Healthcare utilization over study period

Emergency room visits	Total visits: *n* = 64 (37 visits accounted for by 3 participants) Participants that used emergency room: *n* = 14 Mean number of visits across participants: *n* = 2.67 Mode of visits across participants: *n* = 0 Range of visits across participants: *n* = 0–21
Physician visits per person per 30 days	Combined (*n* = 24): mean 1.81 Deceased (*n* = 11): mean 2.53 Alive at study conclusion (*n* = 13): mean 1.20
Hospital length of stay in days per person per 30 days	Combined (*n* = 24): mean 1.20 Deceased (*n* = 11): mean 2.08 Decedents final 30 days (*n* = 11): mean 5.2 Alive at study conclusion (*n* = 13): mean 0.45

Data is reported on 24 of 25 participants. One participant had unreliable healthcare utilization data

### Patient, family and stakeholder satisfaction

Evaluation interviews were conducted with 9 older adults who completed the study and 7 family members. Participants were highly satisfied. They described how the nurse navigator provided support that enabled them to cope with the loss and anxiety that attends chronic illness while navigating a healthcare system fraught with challenges. Participants valued the person-centred approach: adequate time to discuss their concerns, flexible visits in the home, practical action to assist (e.g., picking up equipment), contact with a single credible individual, and an immediate response to a request for assistance. They contrasted this with system-oriented care where they were required to fit into structured, hurried, and overwhelming environments. The personality of the nurse navigator, who was described as personal rather than clinical, was an important factor in the success. Participants described feeling uplifted after the visit – “like a breath of fresh air.”

Participants gave a number of reasons for why they felt they needed a service of this nature including: social and physical isolation; poor access to information; need for emotional support; family conflict; desire to die in their preferred place (either home or institution); and difficulties negotiating physician relationships, particularly when multiple physicians were involved across urban and rural contexts. Benefits of the service included assistance with preparing for what was to come; negotiating family and healthcare system conflict; providing information and assistance with decision-making; normalizing experiences; providing hope; attenuating the burden on family and friends; identifying and obtaining available resources; and having a continuing presence of emotional support. Participants described two challenges with the service. At times they were reluctant to ask for assistance because they did not want to be a “burden”, but participants attributed this to their own way of being rather than any perceptions related to the nurse navigator. Further, in the latter stages of illness they experienced a loss of privacy as a result of the number of individuals visiting in the home.

Evaluation interviews were conducted with the program advisory group (*n* = 6), clinical team (*n* = 3) and a community physician who had referred participants to the service. Respondents were unanimous in the belief that the service filled an important role for this underserved population, citing similar benefits to those mentioned by participants such as education, advocacy, instrumental assistance, support with decision-making, and the person-centred approach. They suggested that the success of the service was largely related to the credibility of the nurse navigator who was known and trusted in the community. In light of the multiple benefits, they recommended that the service be promoted broadly within the community and connections made to other community-based services for older adults. A critique of the program was its lack of visibility within the larger community. The nurse navigator engaged in strategies to promote visibility, but once the pilot reached capacity, it was difficult for her to continue this work while conducting clinical visits. Keeping physicians informed about the service was challenging. Physicians received faxes every 3 months about the progress of their patients; however, at least two physicians could not remember having seen any reports. The need for innovative ways to communicate more strategically with physicians was an important finding.

The lack of formal integration with healthcare services was perceived as the most problematic aspect of this service. Health authority representatives who served on the Advisory Committee were supportive of this person-centred approach for older adults, but were concerned about the cost of the intervention and its potential overlap with other healthcare services once participants were actively dying and more in-home services were available. Overlap did not occur to any substantial degree in this project. With her longstanding work history in the community, the nurse navigator was careful to respect boundaries between her role and that of the community nurses. This was not difficult to do in this context, because community nursing typically became involved when specific tasks, such as wound care or pain management, were required. These tasks were not a substantive part of the navigator’s role. However, it is likely that the navigator’s surveillance and advocacy role enabled clients to gain early and increased access to community care nursing and other relevant services. Overall, the service was viewed as the “ideal” of care, but sustainability was a concern.

## Discussion

The aim of this study was to pilot a nurse-led navigation service to provide early palliative support for rural older adults and family members living at home with advancing chronic illness. Research questions for this pilot were aimed at evaluating feasibility, acceptability, and preliminary outcomes. The service was feasible, acceptable, and effective in meeting the needs of this population. Preliminary outcomes indicated that healthcare utilization was minimal and appropriate, client satisfaction was high, the use of the MQOL was feasible and yielded stable client scores over time. The use of the CSNS was challenging for caregivers of this early palliative population.

Once recruited, older adults remained on the service for prolonged periods of time with no study attrition. Only 28 of the 553 in-person visits were cancelled over the 2-year period. All participants who expressed a preferred place of death were able to die in that preferred place. Emergency room usage for this group of participants was minimal, appropriate (i.e., conditions could not have been managed at home), and lower than Canadian averages. A recent study examining the relationship of home care nursing and emergency department usage in Ontario, Canada indicated that 85% of decedents had an emergency department visit in the last 6 months of life [35]. Four of the 11 participants who died while on our study had no emergency room usage, and a further three participants who died used it only once. These findings are important because Canadian research comparing healthcare utilization between rural and urban populations indicates that rural costs are greater for emergency room usage [36, 37].

Qualitative evaluation data indicated that older adults and family members were highly satisfied with the service, citing specific benefits such as provision of information, psychosocial support, practical assistance, and assistance with decision-making. They particularly valued the person-centred nature of the service whereby someone whom they knew and trusted came into their home to assist at their discretion and on their schedule. The need for a service of this nature was supported by participants ‘longitudinal QOL scores. Similar to the findings of other studies with this population [12, 13, 15, 38], the burden of chronic illness had a significant effect on QOL. Many participants reported longitudinal health-related QOL scores that would match the descriptor of a “bad day,” and the most prevalent troubling symptoms were identical to those reported in other literature: pain, fatigue, sleep disturbance and gastrointestinal difficulties [34]. This early palliative population is coping with similar challenges to those who are actively dying, but without the important home supports that are available in rural areas for those closer to death. Our findings further attest to the urgent need to close the gaps in care for this population. In future studies it would be important to adapt the service to the needs of those living with dementia.

However, the question arises of why the longitudinal QOL scores did not reflect the improvements in quality of life that participants reported in the interviews. Failure to achieve increased QOL scores as a result of home-based palliative services have been reported elsewhere in the literature [39]. Perhaps stabilization of scores was in itself a positive outcome in this population who are experiencing declining health. The increasing unmet need scores reported by the families of patients who died also suggest a level of complexity for the families of those who are experiencing declining health. However, it is also possible that the service provided an intervention that may not be reflected on a multi-dimensional QOL instrument. This explanation is supported by the interview data. Participants reflected on how the presence of the nurse navigator provided support amidst challenges, which qualitatively changed their perception of the challenges, but not the challenges themselves. They still struggled with chronic illness – the healthcare system was still complex and challenging. Using the quality of life instrument as a patient-reported outcome measure was important because it provided a structured means through which adults could communicate their challenges. However, not all participants agreed that it was useful. In future it will be important to select evaluation measures that are sensitive to how participants experienced this intervention.

The many different types of healthcare providers involved with this sample of individuals over this 2-year period support the importance of having a navigator who can enable older adults to understand options and negotiate best-fit care. Her interventions crossed domains of care (e.g., physical to spiritual) and helped to bridge the silos of health and social care in the community. With broad knowledge of the resources available in the community, and of the existing social connections, she was able to mobilize support in an integrated way. An approach that uses caring connections to facilitate access to community supports has strong evidentiary support [17, 18] and the individualized nature of the intervention illustrates best practices for this population [9, 40], particularly as it pertains to end-of life communication [41]. The nurse navigator ‘s long history of working as a nurse within this community, and participant’s relational continuity with one nurse, were important factors in implementation.

In summary, evaluation of the service was positive and data collected illustrate the potential of a navigator to better meet the needs of this growing population. Further research is required to address issues of sustainability and potential applicability of the role for urban contexts. Sustainability issues are related to better integration with formal healthcare services and to the cost of the service. In a previous publication, we described how this service was planned as part of a community-based intervention that was guided by key stakeholders from municipal government, healthcare leaders, and patients and families, with the intent that the service would be sustained and well integrated into the community [25]. This goal was not realized, perhaps in part, because there was significant turnover in leaders in the healthcare region during this time, and it was difficult to produce a cost/benefit analysis within a single pilot site. However, sustainability of the project was achieved through additional research funding in which we piloted a navigation model that was developed around volunteer/healthcare provider partnerships [42]. Clients who finished the study described in this paper had the option of re-enrolling on the new service. In the new service they received visits every 2 weeks by a volunteer, who received additional training in navigation, and visits every 3 months by the nurse navigator. The nurse navigator role changed from providing direct care to providing oversight and mentorship to volunteers. Whether implemented independently, or partnered with volunteers, a nurse-led navigation service can meet the unique needs of rural communities by enhancing support and access in the face of limited healthcare resources.

Although formal integration was not achieved in this study, it would not be difficult to do so. As this is an early palliative population, the most logical connection is to the primary care system. At this early stage, many adults are not yet being seen by home nursing services. Therefore, family physicians, particularly those who work within integrated care networks that include nurses, would be the most appropriate connection for integration. This would support physicians’ capacities to provide better home-based care and facilitate an early palliative approach to care without the requirement of a formal palliative designation and its attending stigma [43].

The cost of the service may also be a significant sustainability issue for constrained healthcare budgets. Based upon a biweekly visit schedule, a nurse navigator can support between 25 and 30 clients. In future, we recommend flexible visit schedules of 1–4 weeks, depending upon client acuity, and nurse navigator caseloads of 40 clients. This would extend the capacity of the service beyond what was obtainable in this pilot. Further, in light of the limited use of the 24/7 call line during this pilot, we recommend finding more cost effective ways of providing this out of hours service. Preliminary findings from this pilot suggest that a nurse navigator may reduce other healthcare utilization costs; however, this needs to be tested more rigorously in future research. It is feasible that a nurse navigator, through advanced care planning and active management at home, can reduce emergency room usage, hospital admissions, and primary care physician visits. Similarly, an important role for the nurse navigator is helping clients to identify available benefits and cost-effective alternatives to care, therefore, cost savings to clients and families should be considered as well.

When considering scaling up and adapting this service to other contexts, there are important factors to consider. Implementation in rural contexts should follow a similar community-based approach, which draws upon existing palliative champions who are well known in the community, to maximize the potential for sustainability and effectiveness [44, 45]. Although this service was designed to meet the unique needs of rural communities, the nurse navigator role could be adapted to urban contexts. The relatively resource-rich nature of urban communities may mean that the navigator focuses more on integrating services than on solving the challenges of limited services. Further, when the navigator is not a person known within the community, older adults may be less willing to have someone come into their home. This would require careful consideration of how to best recruit older adults onto the service.

## Conclusion

Using a nurse navigator to facilitate a palliative approach to care for rural older adults living with advanced chronic illness is a promising innovation for meeting the needs of this population. The person-centred approach, whereby complex problems were addressed in an individualized and incremental manner, is typical of best practices for this population. The service provided continuity for patients and families who were seeing multiple healthcare providers and resulted in a high degree of satisfaction from participants. Further research needs to be done to determine how to integrate the model into currently existing services to enhance sustainability and to evaluate outcomes on a broader scale.

## Abbreviations

MQOL: McGill Quality of life questionnaire
QOL: Quality of life

## References

[1] Albrecht H, Comartin J, Valeriote F, Block K, Scarpaleggia F. Not to be forgotten: care of vulnerable Canadians. 2011. https://www.mcgill.ca/palliativecare/files/palliativecare/parliamentary_report_eng_dec_2011.pdf. Accessed on 31 Jan 2017.

[2] Canadian Medical Association. A policy framework to guide a national seniors strategy for Canadians. 2015. https://www.cma.ca/Assets/assets-library/document/en/about-us/gc2015/policy-framework-to-guide-seniors_en.pdf. Accessed 1 June 2017.

[3] Stajduhar K (2011). Chronic illness, palliative care, and the problematic nature of dying. Can J Nurs Res.

[4] Haggerty JL. Ordering the chaos for patients with multimorbidity. Br Med J (Clin Res Ed). 2012;345:e5915. 10.1136/bmj.e5915 22960377

[5] Moss AH, Lunney JR, Culp S, Auber M, Kurian S, Rogers J, et al. Prognostic significance of the “surprise” question in cancer patients. J Palliat Med. 2010;13:837–40. 10.1089/jpm.2010.0018 20636154

[6] Shadd JD, Burge F, Stajduhar KI, Cohen SR, Kelley ML, Pesut, B. Defining and measuring a palliative approach in primary care. Can Fam Physician. 2013;59:1149–50. PMC3828085 24235182

[7] Stajduhar K, Taylor C. Taking an “upstream” approach in the care of dying cancer patients: the case for a palliative approach. Can Oncol Nurs J. 2014;24:144–53. 25189052

[8] Burge F, Lawson B, Mitchell G (2012). Multimorbidity: when and how to take a palliative approach to care. BMJ.

[9] Gridley K, Brooks J, Glendinning C (2014). Good practice in social care for disabled adults and older people with severe and complex needs: evidence from a scoping review. Health Soc Care Community.

[10] Sawatzky R, Porterfield P, Lee J, Dixon D, Lounsbury K, Pesut B, et al. Conceptual foundations of a palliative approach: a knowledge synthesis. BMC Palliat Care. 2016;15:1–14. 10.1186/s12904-016-0076-9 PMC4715271 26772180

[11] Mason B, Epiphaniou E, Nanton V, Donaldson A, Shipman C, Daveson BA, et al. Coordination of care for individuals with advanced progressive conditions: a multi-site ethnographic and serial interview study. Br J Gen Pract. 2013;63:e580–8. 10.3399/bjgp13X670714 PMC3722835 23972199

[12] Mason B, Nanton V, Epiphaniou E, Murray SA, Donaldson A, Shipman C, et al. ‘My body ‘s falling apart. ‘ Understanding the experiences of patients with advanced multimorbidity to improve care: serial interviews with patients and carers. BMJ Support Palliat Care. 2016;6:60–5. 10.1136/bmjspcare-2013-000639 25023218

[13] Wajnberg A, Ornstein K, Zhang M, Smith KL, Soriano T (2013). Symptom burden in chronically ill homebound individuals. J Am Geriatr Soc.

[14] Walke LM, Gallo WT, Tinetti ME, Fried TR (2004). The burden of symptoms among community-dwelling older persons with advanced chronic disease. Arch Intern Med.

[15] Eckerblad J, Theander K, Ekdahl A, Unosson M, Wirehn A-B, Milberg A, et al. Symptom burden in community-dwelling older people with multimorbidity: a cross-sectional study. BMC Geriatr. 2015;15:1. 10.1186/1471-2318-15-1 PMC4292813 25559550

[16] National Seniors Council. Report on Social Isolation of Seniors. 2014.https://www.canada.ca/en/national-seniors-council/programs/publications-reports/2014/social-isolation-seniors.html. Accessed 1 June 2017.

[17] Murray SA, Boyd K, Kendall M, Worth A, Benton TF, Clausen H. Dying of lung cancer or cardiac failure: prospective qualitative interview study of patients and their carers in the community. Br Med J (Clin Res Ed). 2002;325:929. 10.1136/bmj.325.7370.929 PMC130056 12399341

[18] Gallagher LP, Truglio-Londrigan M (2004). Community support: older adults ‘ perceptions. Clin Nurs Res.

[19] Booth S, Fallon M, Hollis G (2016). Rhetoric and reality - matching palliative care services to meet the needs of patients of all ages, with any diagnosis. Palliat Med.

[20] Williams AM, Kulig JC, Kulig JC, Williams AM (2012). Health and place in rural Canada. Health in rural Canada.

[21] Pitblado JR, Kulig JC, Williams AM (2012). Geographical distribution of rural health human resources. Health in rural Canada.

[22] Wilson DM, Thomas R, Kovacs Burns KK, Hewitt JA, Osei-Waree J, Robertson S (2012). Canadian rural-urban differences in end-of-life care setting transitions. Glob J Health Sci.

[23] Pesut B, Bottorff JL, Robinson CA (2011). Be known, be available, be mutual: a qualitative ethical analysis of social values in rural palliative care. BMC Med Ethics.

[24] Pesut B, Robinson CA, Bottorff JL (2014). Among Neighbours: an ethnographic account of responsibilities in rural palliative care. Palliat Support Care.

[25] Pesut B, Hooper B, Robinson C, Bottorff J, Sawatzky R, Dalhuisen M (2015). Feasibility of a rural palliative supportive service. Rural Remote Health.

[26] Fillion L, Cook S, Veillette AM, Aubin MI, de Serres M, Rainville FO, et al. Professional navigation framework: elaboration and validation in a Canadian context. Oncol Nurs Forum. 2012;39:E58–69. 10.1188/12.ONF.E58-E69 22201669

[27] Case MAB (2011). Oncology nurse navigator: ensuring safe passage. Clin J Oncol Nurs.

[28] Duggleby W, Anderson J, Baxter S, Berry P, Cooper D, Fainsinger R, Fassbender K, Fraser K, Gayman K, Ghosh S, Goodridge D, Hallstrom L, Kaasalainen S, Kary S, Keating N, Kenmore K, MacLeod R, Mann A, Nekolaichuk C, Pesut B, Peterson Fraser M, Robinson C, Santos Salas A, Swanson S, Swindle J, Watanabe S, Watson L, Whitfield K, Williams A, Woytkiw T. Which way from here? Navigation Competencies for the Care of Older Rural Adults at the End of Life. 2014. http://www.nurs.ualberta.ca/livingwithhope/library/Which%20Way%20from%20Here%20Final%20Report.pdf. Accessed 20 Jan 2017.

[29] Cohen SR, Mount BM, Strobel MG, Bui F (1995). The McGill quality of life questionnaire: a measure of quality of life appropriate for people with advanced disease. A preliminary study of validity and acceptability. Palliat Med.

[30] Cohen SR, Mount BM, Bruera E, Provost M, Rowe J, Tong K (1997). Validity of the McGill quality of life questionnaire in the palliative care setting: a multi-centre Canadian study demonstrating the importance of the existential domain. Palliat Med.

[31] Dumont S, Turgeon J, Allard P, Gagnon P, Charbonneau C, Vezina L (2006). Caring for a loved one with advanced cancer: determinants of psychological distress in family caregivers. J Palliat Med.

[32] Hileman JW, Lackey NR, Hassanein RS (1992). Identifying the needs of home caregivers of patients with cancer. Oncol Nurs Forum.

[33] Sandelowski M. What's in a name? Qualitative description revisited. Res Nurs Health. 2010;33:77–84. 10.1002/nur.20362 20014004

[34] Cohen RS, Mount BM (2000). Living with cancer: “good” days and “bad” days--what produces them? Can the McGill quality of life questionnaire distinguish between them?. Cancer.

[35] Seow H, Barbera L, Pataky R, Lawson B, O ‘Leary E, Fassbender K, et al. Does increasing home care nursing reduce emergency department visits at the end of life? A population-based cohort study of cancer decedents. J Pain Symptom Manag. 2016;51:204–12. 10.1016/j.jpainsymman.2015.10.008 26514717

[36] Dumont S, Jacobs P, Turcotte V, Turcotte S, Johnston G (2015). Palliative care costs in Canada: a descriptive comparison of studies of urban and rural patients near end of life. Palliat Med.

[37] Wang H, Qiu F, Boilesen E, Nayar P, Lander L, Watkins D, et al. Rural-urban differences in costs of end-of-life care for elderly cancer patients in the United States. J Rural Health. 2015;32:353–62. 10.1111/jrh.12160 26586101

[38] Higginson IJ, Gao W, Saleem TZ, Chaudhuri KR, Burman R, McCrone P, et al. Symptoms and quality of life in late stage parkinson syndromes; a longitudinal community study of predictive factors. Plos One. 2012;7:1–11. 10.1371/journal.pone.0046327 PMC3492372 23144781

[39] Horton R, Rocker G, Dale A, Young J, Hernandez P, Sinuff T (2013). Implementing a palliative care trial in advanced COPD: a feasibility assessment (the COPD impact study). J Palliat Med.

[40] Arbaje AI, Kansgara DL, Salanitro AH, Englander HL, Kripalani S, Jencks SF, et al. Regardless of age: incorporating principles from geriatric medicine to improve care transitions for patients with complex needs. J Gen Intern Med. 2014;29:932–9. 10.1007/s11606-013-2729-1 PMC4026496 24557511

[41] Barnes S, Gardiner C, Gott M, Payne S, Chady B, Small N, et al. Enhancing patient-professional communication about end-of-life issues in life-limiting conditions: a critical review of the literature. J Pain Symptom Manag. 2012;44:866–79. 10.1016/j.jpainsymman.2011.11.009 22819438

[42] Pesut B, Duggleby W, Warner G, Fassbender K, Antifeau E, Hooper B, et al. Volunteer navigation partnerships: piloting a compassionate community approach to early palliative care. BMC Palliat Care. 2017. doi:10.1186/s12904-017-0210-3. 10.1186/s12904-017-0210-3 PMC5496423 28673300

[43] Hui D, De La Cruz M, Mori M, Parsons HA, Kwon JH, Torres-Vigil I, et al. Concepts and definitions for “supportive care,” “best supportive care,” “palliative care,” and “hospice care” in the published literature, dictionaries, and textbooks. Support Care Cancer. 2013;21:659–85. 10.1007/s00520-012-1564-y PMC3781012 22936493

[44] Kelley ML. Developing rural communities' capacity for palliative care: a conceptual model. J Palliat Care. 2007;23:143–53. 18069435

[45] Kelley ML, Williams A, DeMiglio L, Mettam H (2011). Developing rural palliative care: validating a conceptual model. Rural Remote Health.

[46] Brazil K, Kaasalainen S, Williams A, Dumont S (2014). A comparison of support needs between rural and urban family caregivers providing palliative care. Am J Hosp Palliat Care.

